# Public School Trauma Intervention for School Shootings: A National Survey of School Leaders

**DOI:** 10.3390/ijerph18157727

**Published:** 2021-07-21

**Authors:** Bree Alexander

**Affiliations:** Social Work Department, Texas A&M University-Texarkana, Texarkana, TX 75503, USA; balexander@tamut.edu

**Keywords:** trauma, public schools, trauma intervention, school shootings, K-12 schools, trauma-informed

## Abstract

Trauma intervention in United States’ (U.S.) public schools is varied. The occurrence of public-school shootings across the U.S. elicits questions related to how public schools currently address and provide resources related to trauma for employees and students. A randomized, national survey of public-school teachers, guidance counselors, and administrators was conducted to gather information on public-school preparedness for response to trauma. Findings indicated that only 16.9% of respondents indicated their schools have trauma or crisis plans that address issues related to school shootings. Furthermore, public schools use a variety of strategies to address trauma, but teachers, guidance counselors, and administrators were often unsure about the effectiveness of these trauma interventions in the event of school shootings. Implications for findings suggest methods to enhance next steps in the area of trauma response to school shootings.

## 1. Introduction

Among public schools in the United States (U.S.), school leaders such as teachers, guidance counselors, and administrators serve on the front lines of student and school needs. Literature indicates that during the typical academic year, school leaders manage school and student needs including but not limited to student academic performance, school activities and events, school district performance, and student mental and psychological health [1]. The issue of addressing trauma, particularly trauma following school shootings is no exception.

There is no universally accepted definition of a school shooting or mass school shooting and these terms are viewed as different phenomena in this area of research. In fact, there is much controversy on the number and trends in school shootings depending on the definitions used. For instance, Peterson and Densely [2] indicate there have been 12 mass school shootings since 1989 based on a definition from the Congressional Research Service which states a mass shooting is:

“a multiple homicide incident in which four or more victims are murdered with firearms, not including the offender(s), within one event, and at least some of the murders occurred in a public location(s) in close geographical proximity (e.g., workplace, school, restaurant or other public settings), and the murders are not attributable to any other underlying criminal activity or commonplace circumstance (armed robbery, criminal competition, insurance fraud, argument or romantic triangle) (para 3)”

However, the K-12 school shooting database indicates there have been 177 active school shooting incidents since 1970 based on the following definition: “a gun is brandished, is fired, or a bullet hits school property for any reason, regardless of the number of victims (including zero), time, day of the week, or reason [3].” Though media coverage of public-school shootings in the U.S. has been amplified in the last 20 years creating the appearance of more frequent and consistent incidents despite school shooting occurrences being statistically rare [4], trauma in the aftermath of these incidents poses a very real concern for many schools across the nation. Regardless of how a school shooting is defined, the initial impact of widespread fear and panic contributes to subsequent trauma symptoms, which is the primary focus of this research. Thus, the definition of school shooting as it relates to this research is “a gun is brandished, is fired, or a bullet hits school property for any reason, regardless of the number of victims (including zero), time, day of the week, or reason” [3].

Negative consequences related to school shootings include trauma symptoms such as posttraumatic stress disorder (PTSD), posttraumatic stress symptoms, major depression, anxiety, and mood disorders which can be manifested in many ways including mental intrusions, flashbacks, sleep problems and/or nightmares, and hypervigilance [5], making trauma intervention an important issue for public schools in the U.S. Common methods of trauma intervention in schools have in the past included school-based mental health services on campus [6]. However, recently, addressing student and employee trauma in schools has increased in awareness and need resulting in more trauma-informed programs and models for schools [7]. Many schools in the U.S. have adopted trauma-informed strategies to help address student behavior and issues stemming from traumatic experiences [8]. One common trauma-informed model used in schools is Positive Behavioral Interventions and Supports (PBIS), which adds trauma-informed direction and progressive intervention to a holistic support approach for students [7,9,10]. However, it is unclear if these strategies are being used in all public schools and what strategies are being used altogether. Additionally, it is unclear if such strategies are sufficient in addressing trauma related to public school shootings.

The purpose of this study was to conduct a national survey of public-school teachers, guidance counselors, and administrators in any U.S. public school to explore the need for resources related to trauma and the role of public schools in managing the psychological effects of school-based traumatic incidents such as school shootings. The following research question was addressed:

1. What programming and resources are U.S. public schools providing (or providing access to) for students, public school teachers, guidance counselors, and administrators related to school shootings and/or surviving school shootings?

## 2. Literature Review

U.S. public schools and education systems have undergone criticism related to the management of trauma or perhaps lack thereof, in the aftermath of public-school shootings [11]. The effects of trauma related to experiencing a school shooting can put individuals at risk for mental health disorders such as depression, anxiety, post-traumatic stress disorder (PTSD), and acute stress disorder as well as sleep disturbances, emotion dysregulation, poorer academic performance and classroom behaviors, lower grade point average, increased school absences, relationship difficulties, decreased work satisfaction, and substance abuse [12,13,14,15,16,17]. Research to examine public school recovery measures related to school shootings has led to possible methods of providing support to students but lacks information regarding ways to support faculty and staff or commonalities in plans and/or programs used among public schools across the U.S. [16]. Due to the significance of the psychological effects of public-school shootings, research warrants more answers related to how to better manage trauma related to public school shootings. This gap in the literature suggests that barriers may exist for methods to manage the impact of trauma in schools in general, but especially following school shootings.

### 2.1. Public-School Response to Trauma

Schools are appropriate and practical settings to help students and school employees recover from tragedy as reported by the National Institute of Mental Health: “when violence or disaster affects a whole school or community, teachers and school administrators can play a major role in the immediate recovery process by providing specific structured and semi-structured activities” [14] (Para 2) [18]. Instances of school shootings have increased school administrators’ awareness of the need for crisis plans and according to the U.S. Government Accountability Office [19] approximately 95% of schools nationwide have a crisis plan in place [20]. However, though “many schools have well-developed emergency management plans, an important piece consistently missing from plans is post-crisis recovery” [14] (para 5). Following school crises, interventions can range from individual counseling to large debriefing groups and/or assemblies [21]. However, school leaders (e.g., public school teachers, guidance counselors, and administrators) report that public schools are struggling with adequate responses to trauma in the aftermath of traumatic events such as school shootings [9]. Despite providing access to mental health resources for students, faculty, and staff members, many report that public schools are missing the mark in providing adequate care related to trauma for those who have experienced school shootings [9]. “Attention to childhood trauma and the need for trauma-informed care has contributed to the emerging discourse in schools related to teaching practices, school climate, and the delivery of trauma-related in-service and preservice teacher education” [22] (p.423). This may help address the gap in trauma intervention at public schools in the aftermath of school shootings.

### 2.2. Trauma-Informed Practices

The National Child Traumatic Stress Network posits that creating a trauma-informed system involves “one in which all parties recognize and respond to the impact of traumatic stress” [23] (Para1). Trauma-informed care systems can vary from setting to setting. However, these approaches strengthen resilience and protective factors of those impacted by trauma. This mitigates the impact of trauma on other systems (i.e., family, school, etc.), emphasizing continuity of care and collaboration, and maintaining an environment that addresses and minimizes trauma triggers and increases wellness [23]. Though all these components are important to the trauma-informed response, priorities lie in activities that build meaningful partnerships at the individual and organizational levels and address the intersections of trauma and its compounding impact on traumatized individuals [23]. The use of trauma-informed care in schools specifically regarding school shootings raises many questions such as how practices may change over time and how practices may vary in schools with shooting history and schools without shooting history. When dealing specifically with school shooting survivors, trauma exposure is, unfortunately, a substantive issue, making trauma-informed care an appropriate intervention tool for public schools to use to address trauma symptoms.

### 2.3. Trauma-Informed Methods and Practices in Schools

In many states, trauma-informed practice is connected to social and emotional learning, school safety, school discipline, and/or Positive Behavior Interventions and Supports (PBIS) [22]. Schools may offer resources to teachers and staff members such as toolkits, research or practice briefs, guidebooks, PowerPoint slides, and online training and learning modules [22]. Many approaches focus on the individual student or teacher–student interaction and how it can be adapted to support student emotional, social, and academic growth following trauma exposure. For a school to be trauma-informed, there needs to be several components and/or foundational principles addressed including building a sense of community, social and emotional connectedness, facilitating knowledge of prevalence and impact of trauma, building capacity of educators and caregivers, empowerment and resiliency, and promoting mindset change by addressing causes of behavior and social justice [24].

However, implementation of trauma-informed methods at school has been slow to develop across U.S. public schools despite recommendations. Contexts, where trauma-informed practices are most heavily promoted, include high poverty schools, alternative programs, large urban districts, and rural settings [22]. These trends are disheartening since research indicates that trauma-informed methods can be applied to any program, organization or system that (1) realizes the widespread impact of trauma and understands potential paths of recovery, (2) recognizes the signs of symptoms of trauma in the clients, families, staff, and others involved with the system, (3) responds by fully integrating knowledge about trauma into policies, procedures, and practices, and (4) seeks to actively resist re-traumatization [25]. By these standards, all public schools could benefit from use of trauma-informed practices. Additionally, school shooting incidents have historically occurred in schools that are not associated with high poverty, alternative programs, or large urban districts (where trauma-informed practices are heavily promoted) leaving them vulnerable to inadequate trauma management following a shooting. However, the more pertinent task seems to lie in determining how to implement and evaluate trauma-informed methods in schools that have experienced school shootings and if this is an effective trauma response plan for them.

### 2.4. Trauma-Informed Methods and Practices in Schools with Shooting History

Trauma-informed supports are, arguably, equally as important for school staff when discussing school shooting scenarios. Application of trauma-informed methods in public schools, however, may be difficult when specifically related to school shootings because schools would need to address the trauma for faculty members in addition to students. Trauma-informed school methods in response to school shootings should consider how such methods will affect teachers and staff members who may be experiencing similar trauma symptoms. One of the nuances of managing trauma in the aftermath of public-school shootings involves attending to the trauma of both students and faculty and staff members [9]. While trauma-informed school methods are beneficial to students who have experienced trauma, one must question, in scenarios related to public school shootings, if a trauma-informed model that often relies heavily on facilitation from teachers and school staff members is beneficial for their healing and potential trauma symptoms, or how such a model can be adapted so that it is beneficial for all and provides the support needed for faculty/staff. Literature in this area is limited and has yet to address disadvantages or advantages for school faculty and staff administering trauma-informed practices for instances of shared traumatic experiences such as school shootings. Due to the varied methods used by public schools to address trauma and trauma recovery, it is unclear what strategies are being used in schools across the nation, how they relate to school shootings, and how they are perceived by school leaders (e.g., teachers, guidance counselors, and administrators).

## 3. Method

The author posed the following research question: What programming and resources are U.S. public schools providing or providing access to for students, public school teachers, guidance counselors, and administrators related to school shootings and/or surviving school shootings? A cross-sectional design approach was used to develop a questionnaire to emphasize description and exploration of trauma response services and resources currently offered in public schools and attitudes towards these services. Prior to conducting this study, Institutional Review Board (IRB) approval was obtained through Baylor University. A consent statement was presented to participants on the first page of the survey and participants were informed that advancement to the next section of the survey indicated consent. Survey settings were set to ensure that participants were not able to complete the survey more than once. Next, the author analyzed and compiled findings to disseminate among professionals interested in trauma response to public-school shootings.

### 3.1. Participants

The sampling frame included 360,000 public school teachers, 88,000 administrators, and 58,804 guidance counselors, all of which were obtained from a national marketing email listserv. Upon obtaining contact lists in the sampling frame, 500 individuals from each list of eligible public-school employees (i.e., public school teachers, public school administrators, and public-school guidance counselors) were randomly selected for a total of 1500 individuals. The rationale for this sampling frame is related to Rubin and Babbie’s [26] estimation of approximately 50% response rate for online surveys and accounts for a sample size large enough (i.e., 750 participants) to provide national estimates for public schools in the U.S. Participants were randomly selected via a random selection tool within the survey software system (i.e., Qualtrics). Initial invitations that included recruitment contact and a link to participate in the survey were sent out to all teachers, administrators, and guidance counselors in the sample. Multiple contacts included the initial formal invitation email, an initial follow-up reminder email 3 days after the initial formal invitation, subsequent follow-up reminder emails at two weeks, four weeks, six weeks, eight weeks, and nine weeks after the initial follow up which included replacement names from prior nonresponse emails. A final thank you letter was sent for those that participated, with a reminder for those who did not participate to do so. Additionally, subsequent contacts included a link to assess for nonresponse bias. All survey responses were anonymous and no identifying information was obtained.

### 3.2. Assessment and Procedure

The study used an anonymous, online survey to obtain information from participants. The author was not able to identify where any completed questionnaire came from; however, informed consent was connected to the individual’s survey to indicate which surveys would need to be pulled out if participants decided to withdraw from the study. Potential participants were randomly selected from the sampling frame to receive a formal, in-depth invitation letter via their school-related email accounts to complete the anonymous online survey via link. Participants who voluntarily agreed to complete the survey clicked the survey link in their formal invitation which led to the overview of the study describing the study in detail, defining pertinent terms related to the study that were used throughout the survey, and thanking participants for their participation. Participants also reviewed an informed consent prior to completing the survey including potential risks and benefits of the study, anonymity, voluntary participation, protection of human rights, and explanation of their role in the study. Advancement to the next section and completion of the survey indicated consent. Three days after the formal invitation, a follow up email was sent to participants encouraging those that had not participated yet to do so. Following an additional two weeks, another email was sent of a similar nature thanking participants who completed their surveys and encouraging others to participate if they had not. Additional emails were sent at the six week, eight week, and nine week marks for follow up and reminders to complete the survey. Data were collected based on percentage of responses during each wave of reminders. Those whose emails were not working during each wave of contacts were replaced with another name randomly selected from the original sampling frame. To address nonresponse rate bias, a link was provided for those who did not respond to the formal invitation after two emails to assess why survey responses were not provided.

The survey (i.e., the Public-School Trauma Support Assessment) included 25 five-point Likert scale items and 1 open-ended item that were informed by data obtained during a previous study, a five-item PTSD scale for those who had experienced school shootings only, and a demographic section. Some items included examining differences in strategies used among public schools in response to school-based traumatic events and perceived barriers among employees toward implementing a public-school intervention for school-based trauma were from the School Survey on Crime and Safety for Principals [27]. This survey’s use in the study was based on its use in previous national surveys of public schools by the U.S. Department of Education to measure similar concepts such as school violence, although it focuses on the broader topics of crime and safety and does not address trauma support in public schools. The most recent survey sample in 2016 included approximately 3553 public elementary, middle, and high schools nationwide [28]. Specifically, the author used eight questions from this survey on topics related to school mental health services, school practices and programs, staff training and practices, and school security staff. Sixteen questions on topics related to parent and community involvement at school, crime incidents, disciplinary problems and actions, and school characteristics from the School Survey on Crime and Safety for Principals were excluded due to being outside of the scope of this study. An additional 18 questions were developed by the author and added to the survey to assess for trauma support services, public school employee perceptions of such services, and differences among strategies that are reported in the literature as being used in public schools to address trauma in students and employees. Participants would, then, identify strategies for public school efforts toward improvement of trauma response to school shootings via questions provided within the survey. The open-ended question was included in the survey for respondents to discuss various strategies used by their schools to reduce trauma symptoms in individuals. The author also requested basic demographic information including age, gender, ethnicity, job title, years of employment in public schools, years of employment in current school district, and number of public schools employed. Participants were expected to complete the survey in approximately 10–15 min. The data collection process lasted for two and a half months.

### 3.3. Data Analysis

The author calculated effect size using Cohen’s d to determine minimum sample size needed for the study. The results indicated 329 respondents were required to exceed Cohen’s [29] convention for a large effect (*d* = 0.80). Cronbach’s alpha calculation for internal consistency of the survey questions indicated high reliability (*α* = 0.88). Expert panel results determined that survey items captured the intended concept of trauma support in schools. SPSS software was used for analysis of survey responses. Basic descriptive analyses (e.g., frequencies, central tendency) provided the percentage of public schools responding to the survey that have a trauma/crisis plan in place. Additional chi square testing was used to compare the attitudes toward public school intervention in the event of school shootings based on respondent’s position at their school (e.g., teacher, guidance counselor, and administrator). Additionally, basic descriptive analyses and axial coding helped to identify themes in public schools’ strategies to reduce trauma symptoms in students and employees for the open-ended survey item. Alpha was set to 0.05. The author reviewed data for the standard assumptions of each test before conducting any analyses.

## 4. Results

The total sample included 500 public school teachers, 500 guidance counselors, and 500 administrators. The survey response rate was 27.73%. Survey respondents consisted of 416 (303 identified as women, 72 identified as men, 1 identified gender as not important, and 40 gender nonresponses). Roles included 18 respondents identifying as school administrators, 255 as teachers, 90 as guidance counselors, and 53 not identifying their role. The age of participants ranged from 23 to 67 years old (*M* = 38.92, mode = 29). The average length of employment in public schools for participants was 13.14 years (range: 1 year to 53 years of experience; *Mdn* = 11). A total of 96.8% of respondents identified themselves as full-time public-school employees, 2.8% identified as part-time, and 0.3% did not identify employment status. A total of 59% of survey respondents identified as Caucasian, 24.8% identified as African American, 1.7% identified as other race (e.g., Asian, Middle Eastern), 8.2% identified as Hispanic/Latino, and 5.5% of respondents did not identify a race. Survey respondents represented four regions of the United States: Northeast, Midwest, west, and south. There were 132 respondents from the south region, 85 from the west region, 74 from the Northeast region, 84 from the Midwest region and 41 nonresponses. A total of 73% of survey respondents identified as women and 17.3% as male. An additional 12 respondents completed the separate non-response survey instead of the study survey citing lack of time and concern for the study topic as primary reasons for not completing the study survey.

### 4.1. Trauma and/or Crisis Intervention Plans

As shown in Table 1, across all survey respondents, 47.4% percent of respondents agreed or strongly agreed that their school possessed a written trauma and/or crisis plan that describes procedures to be performed in the event of a school shooting. However, only 16.9% were able to agree or strongly agree that their school had a plan that describes trauma intervention strategies to be used in the event of a school shooting. Additionally, 52.5% of respondents were either unsure if their school had a written plan to address school shootings or disagreed altogether that such a plan existed. A total of 83% were unsure or disagreed that their school’s plan included trauma intervention strategies that can be used following a school shooting.

Additionally, 61% of school administrator respondents confirmed their schools have trauma and/or crisis intervention plans compared with 44% of teacher respondents and 48% of guidance counselor respondents suggesting that administrators may be more likely to be knowledgeable about such plans compared with other respondents (i.e., teachers and guidance counselors).

### 4.2. Trauma Intervention Strategies in Public Schools

Differences in strategies used among public schools to reduce symptoms of trauma in the event of a school shooting were determined using an open-ended survey question which prompted respondents to identify strategies and/or methods that their school uses to address trauma (i.e., “Please share any strategies that your school uses that may reduce trauma symptoms in students or teachers in the event of a school shooting”). Additionally, other survey questions addressed the use of strategies commonly viewed as beneficial for trauma intervention used in schools such as the presence of a mental health counselor and police officer on campus. Additionally, 86.1% of respondents “agreed” or “strongly agreed” that their school possesses a mental health counselor on campus and 93.6% “agreed” or “strongly agreed” that there is a police officer on their campus.

Some themes emerged from the open-ended survey data regarding trauma intervention. Respondents identified trauma methods within two main themes: prevention and intervention strategies that are used in their schools. Common prevention strategies included peer mentoring and anti-bullying policies and programs. Common intervention strategies included mental health services and specific trauma-informed strategies such as restorative circles. However, it was noted that anti-bullying policies and mental health services appeared to be the most popular strategies mentioned among respondents. Peer mentoring and restorative circles, although mentioned often, were not mentioned as often compared with the aforementioned.

### 4.3. Attitudes toward Public School Trauma Intervention Following School Shootings

**Figure 1:**
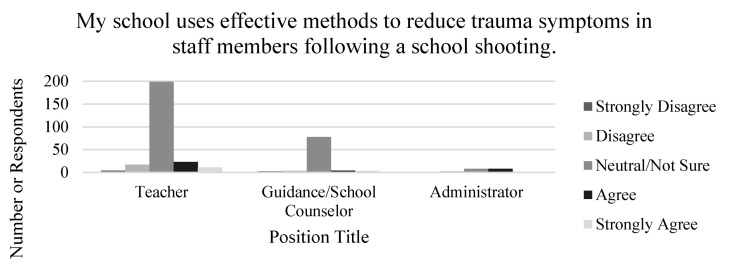

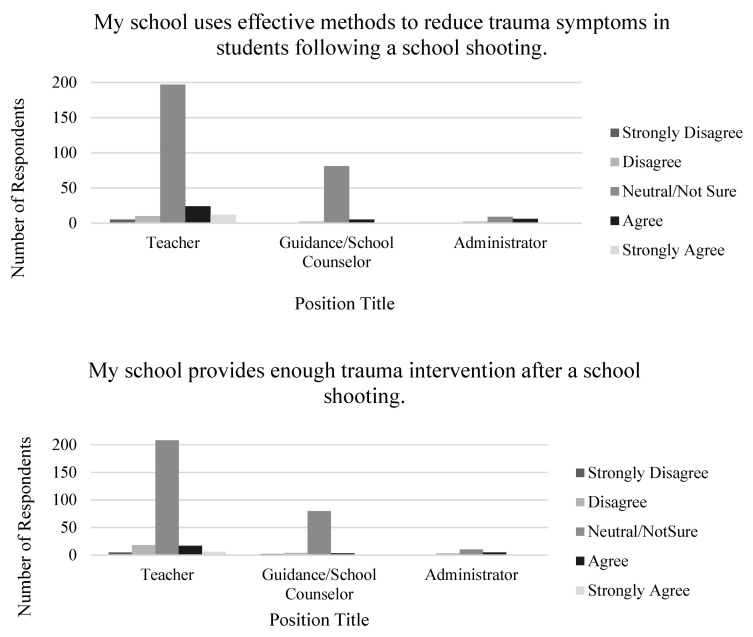
Attitudes toward Public School Intervention.

The difference in attitudes toward public school trauma intervention related to school shootings among teachers, administrators, and guidance counselors was assessed via level of agreement based on the following three survey items: (1) My school uses effective methods to reduce trauma symptoms in staff members following a school shooting, (2) My school uses effective methods to reduce trauma symptoms in students following a school shooting, and (3) My school provides enough trauma intervention after a school shooting. Many survey respondents across each position type reported neutral/unsure attitudes toward public school intervention following school shootings (See Figure 1). Furthermore, 298 survey respondents were unsure if their school does enough intervention related to school shootings.

Figure 1 respondent graphs indicating responses across every position type showed a majority of neutral/not sure responses to questions regarding the effectiveness and sufficiency of trauma intervention strategies used in schools for staff members and students.

A chi-square test of independence was conducted to compare the responses toward public-school trauma intervention strategies based on the respondent’s position at their school. Perception of trauma strategies in public schools was varied among respondents. There was a significant association between respondents’ position at their school and level of agreement regarding effectiveness of trauma intervention in schools for staff members, χ^2^ (12, *n* = 375) = 38.39, *p* = 0.000). There was also a significant association between respondents’ position at their school and level of agreement regarding effectiveness of trauma intervention used in schools for students, χ^2^ (12, *n* = 375) = 28.48, *p* = 0.005). Finally, there was a significant association between respondents’ position at their school and level of agreement regarding if their school provides enough trauma intervention following a school shooting χ^2^ (12, *n* = 375) = 24.85, *p* = 0.016). Specifically, findings indicated that a majority of respondents were unsure if their schools provided effective methods to reduce trauma symptoms in staff members and students.

There was also determined to be a correlation of survey items related to trauma plans and effectiveness of trauma intervention strategies in schools. Specifically, positive responses to survey items about schools having a written plan to address trauma and school shootings were more likely to be associated with positive responses to items related to effectiveness of their school’s trauma strategies, *r* (373) = 0.520, *p* < 0.01. Additionally, respondents who confirmed their school used effective methods of trauma intervention in students and staff members were also more likely to select positive responses to the survey question related to preparedness to manage trauma in a student following a school shooting, *r* (373) = 0.41, *p* < 0.01.

## 5. Discussion

This study focused on how public schools currently address trauma on campus and school leaders’ (e.g., guidance counselors, teachers, and administrators) perceptions of these methods. The magnitude of effect was relatively large, and several outcomes offered insight for public schools and school districts to use in future planning for trauma intervention on campus. The overall findings suggest that public schools across the nation use a range of strategies that could potentially be used to address trauma on campus. Specifically, respondents consistently reported a lack of and/or unawareness of written trauma and/or crisis intervention plans in schools. However, many respondents also reported the presence of an on-site mental health counselor. This could suggest many things including, but not limited to, schools perceiving mental health services to be equivalent and/or superior to trauma and/or crisis intervention plans in schools, school employees being more knowledgeable or aware of school mental health services than school trauma plans, or more barriers associated with having trauma and/or crisis plans versus a mental health counselor. Additionally, respondents appeared to be more sure of services that students had access to or would have access to in the event of a school shooting than they were about services for themselves. Overall, trends and correlations in the survey respondent data could provide invaluable information related to how public schools currently address trauma and potential areas of improvement.

### 5.1. Trauma and/or Crisis Intervention Plans

The presence and/or knowledge of a written plan to address school shootings was shown to be a large indicator in a respondent’s perception of a school’s preparedness for trauma or school shootings. This factor was also a large indicator in the respondent’s individual predicted feelings of self-efficacy and preparedness to help manage student trauma response following a school shooting. This suggests that awareness of a written, formalized plan improves public school employee confidence in the school’s ability to respond to school shootings appropriately. However, in many cases, despite some respondents confirming the presence of a written plan related to school shootings, many of these respondents were either unsure or reported that this plan did not include any specific trauma interventions and/or strategies that should be used following a shooting to address traumatic responses. This pattern of findings was aligned with the predicted direction for all responses suggesting that many schools do not possess a written plan related to procedures following a school shooting and those that do often lack emphasis on trauma intervention. Findings also appeared to lack agreement with previous literature which posits most public schools (approximately 95%) have crisis plans [19,20]. This suggests that the emphasis on survey questions placed on school shootings and trauma intervention may have contributed to this difference and perhaps identifies a gap in existing public-school crisis plans.

### 5.2. Trauma Intervention Strategies in Public Schools

Many survey respondents mentioned trauma intervention strategies including peer mentoring programs, anti-bullying policies and programs, and trauma-informed practices such as restorative circles. This aligns with current trauma literature which states that all of these interventions are beneficial in some way to schools for prevention and intervention related to school shootings [16,30,31,32]. Furthermore, mental health services and access to a police officer on the school’s campus was a common intervention identified by survey respondents. Research indicates that the presence of a school-based mental health counselor improves school climate and other positive outcomes for students, such as school safety and lower rates of suspension and other disciplinary incidents [33,34,35]. However, research suggests the opposite for the presence of police officers in schools. Specifically, literature suggests that having police in schools has not resulted in safer schools and can result in an increase in student referrals to police and student arrests for low level incidents, particularly with students of color [36,37]. Thus, the presence of both of these professions on a school’s campus could have a significant effect on prevention of traumatic events and reduction in trauma symptoms related to traumatic events.

Overall results aligned with previous literature which states that more schools are focusing on use of trauma-informed strategies [22]. However, there was little mention of assessment and/or screening used in schools to identify students in need of more intensive supports which literature suggests is a crucial part of trauma intervention [8,38,39] and psychological first aid training for school employees which can be complementary to other trauma-informed programs, is designed to be used by anyone after crises occur, and supported by the National Child Traumatic Stress Network and the National Center for PTSD [8,40]. Thus, findings suggest a need for improved access to and knowledge of trauma intervention resources. This gap raises concerns about the implications of the lack of identified services in school crisis plans, support in the aftermath of a school shooting, and consideration of faculty and staff members who may also be experiencing trauma and how they might manage such responses while also support students.

### 5.3. Attitudes toward Public School Intervention Following School Shootings

Survey respondents were also overwhelmingly unsure of the effectiveness of trauma strategies used in public schools. Findings in this area suggest that school employees often have an unclear perception of trauma strategies used in public schools either due to the lack of strategies used in schools, lack of knowledge of what these strategies are, could be, and how to apply them or inability to determine effectiveness of said trauma strategies. However, when discussing interventions following school shootings, survey respondents appeared to be clear on the services that would be provided to students in the event of a school shooting. Furthermore, they were less clear on the services that would be provided to them in the event of a school shooting. For example, many respondents acknowledged that they were unsure if mental health services would be provided to them in the event of a school shooting and subsequently acknowledged that their school had not made them aware of where they could potentially find resources or access to resources following a school shooting. This suggests an implicit bias and assimilation to the idea that students and their trauma response are the primary concern following a school shooting. However, research suggests that public school employees such as teachers, guidance counselors, and administrators are also vulnerable to experiencing negative effects following school shootings [41]. Thus, failure to consider faculty and staff trauma and how to manage this is an important factor following school shootings. Implications could include disgruntled school employees, negative attitudes toward the school as an organization, increased risk of mental health related concerns, decreased work satisfaction, increased attrition rates, decreased retention rates, and poor relationships with students.

### 5.4. Implications

Schools are a logical setting for trauma prevention and intervention services. Many schools attempt to address these issues with school-based mental health services, however, research indicates that these services tend to target aggressive and disruptive behaviors rather than internalized issues such as trauma symptoms [16,42,43,44]. This could prove to be a barrier for crisis incidents such as school shootings. Though incidents of school shootings are rare, considering school trauma responses for such a crisis would enhance knowledge of trauma resources and changes that need to be made to improve existing services. Changes within the public school response to trauma will require diligent and intentional efforts on many levels within the school system. Based on survey responses in this study, public schools would benefit from addressing trauma intervention across micro, mezzo, and macro levels to establish a more comprehensive approach to trauma response. For example, interventions at the micro level may include a critical evaluation of trauma assessment tools used by schools to determine whether the tool reflects the principles of trauma-informed care and are supported by evidence or the implementation of use of such a tool, if one is not already being used. Trauma-informed care and counseling would also be included in micro level interventions. However, research is still needed on best practices for school shooting trauma. Interventions at the mezzo level might include facilitating separate support groups for students and school employees to build safety, collaboration, empowerment, and systems of support [20]. Finally, interventions at the macro level might include school representatives participating in a trauma or crisis task force in the local community to engage in response efforts and/or advocating with state legislators for access to resources to address school trauma response and its impact on schools and their surrounding communities. Furthermore, trauma-informed models in schools will need to address the widely varied trauma intervention responses among public schools. Trauma literature identifies a variety of trauma-informed methods appropriate for use in schools, however, future research should address the potential benefits of having district, state, or nation-wide recommendations or requirements for such models. Recommendations or requirements should be based on meta-analyses or research conducted with specific trauma-informed models in schools.

### 5.5. Limitations

This study raises many significant questions for future research. The current research question narrowed the scope of scholarly insight on the topic and though this was the intention to gather foundational research, future research would benefit from broader research inquiries on the topic. The practicalities of providing trauma training to staff such as grants to purchase materials and consultants to adapt resources to a particular school, the impact of using these resources and the resilience they build, the varying needs of students versus school employees following a school shooting, and the effectiveness of trauma-informed practices in school shooting scenarios are all areas for future research consideration. Additionally, this study does not include a comparison of psychometric properties of aforementioned trauma tools and interventions, and thus, cannot contribute to discussion of the superiority of any particular measures. Discrepancies in ratings of effectiveness of trauma intervention strategies across respondents (e.g., administrators, guidance counselors, and teachers) were common. It is unclear, however, how to interpret the lack of group differences in some self-reported outcomes in this study. This pattern of findings may reflect limitations in the assessment measures, in respondent comprehension, or in respondent willingness to disclose information regarding their school. Methodological limitations of this study may include small sample size compared with sample frame and limitations to the randomization process. However, I noted an increase in responses following each reminder email suggesting that a longer data collection period with additional reminder emails would have likely resulted in a larger sample size. The demographics of the sample also indicated disproportionate representations of gender and race in survey respondents as well as the inherent roles and regions of survey respondents potentially having significant differences in their experiences and access to trauma resources and services. Furthermore, differences in response rates between groups (e.g., number of teacher respondents versus administrator respondents) could have led to statistical biases when comparisons were made among groups. Future research may benefit from combining groups with unequal sample sizes for comparisons with less risk for bias. Ultimately, response rates could have been affected by several variables including the roles of the respondent in the school, the region the respondent was in, structure of the respondent’s school day, etc. Unfortunately, the nature of the study offered very little information about those who did not respond or complete the nonresponse survey making determining the extent to which non-respondents are different from respondents difficult. Additionally, schools may be reluctant to invest time and money in trauma intervention for the aftermath of school shootings versus prevention efforts. Despite these limitations, the study suggests that further research in this area is warranted.

## 6. Conclusions

The findings of this study offer insight from public school leaders on how schools are currently addressing trauma in schools including significant gaps in both interventions and research for effectiveness. One glaring topic is the impact of school shooting trauma on staff and faculty who are then providers of care and comfort while managing their own responses even as all of the school requirements continue. This information provides baseline data on public school trauma awareness, trauma intervention, and, in some cases, perception of intervention effectiveness. This knowledge can assist in identifying trends related to addressing trauma in public schools, particularly following school shootings, and determining what needs to be done to create an effective and perhaps systematic response to trauma related to school shootings in public schools. Findings indicate that the lack of trauma response and/or intervention plans that address needs following school shootings in public schools further perpetuates unpreparedness for traumatic events that may occur on or near campus. These challenges exacerbate existing inadequate knowledge of education professionals related to the effects of trauma and how to appropriately respond when it has occurred. Further, the lack of knowledge and/or communication of trauma response and/or intervention plans yields similar consequences.

Overall, the history of public school responses to crises suggests that there is capacity for growth in the area of trauma response. Education professionals have demonstrated commitment to improving trauma response in schools that involve a balance among prevention, intervention, and reaction [45]. In fact, school professionals may already be laying the groundwork for trauma-informed environments and improved crisis response by promoting effective coping to students through formal instruction in life skills and building rapport and trust with students while modeling appropriate ways of expressing feelings. Many of these tactics would enhance next steps in the area of trauma response to school shootings.

## Figures and Tables

**Table 1:** Trauma Intervention Plans.

Level of Agreement	Strongly Disagree	Disagree	Not Sure/Neutral	Agree	Strongly Agree
Item 4					
My school has a written plan that describes procedures to be performed in the event of a school shooting.	1%	36.6%	14.9%	31%	16.4%
Item 5					
My school has a plan that describes trauma intervention strategies that can be used following a school shooting.	3.4%	54.4%	25.2%	10.3%	6.6%

## Data Availability

Data presented in this study are available on request from the corresponding author. The data are not public available due to privacy/confidentiality of research participants.
